# Dream-Thought and Dream-Life

**Published:** 1862-04

**Authors:** 


### MEDICAL CRITIC

AND

### PSYCHOLOGICAL

### JOURNAL.

---

APRIL, 1862.

---

### Art. I.—DREAM-THOUGHT AND DREAM-LIFE.

"We are somewhat more than ourselves in our sleep, and the slumber of the body seems to be but the waking of the soul. It is the litigation of our sense but the liberty of reason, and our waking conceptions do not match the fancies of our sleep."—Sir Thomas Browne. *Religio Medici,* Part II. p. 206.

Dreams are continuous thought. They form a state connecting two periods of more perfect attention, vigilance, and responsibility, during which there occurs a repetition, a recombination of former impressions, and occasionally such a condition of consciousness as appears a discovery and a development. In this state all the ordinary or normal processes and laws of thought may be detected; modified by the suspension or enfeeblement of the operation of the external senses, and of that attention which the impressions upon these senses rouse and sustain in activity; and indirectly, by the physical condition which induces and accompanies sleep so long as that continues.

There are, then, two aspects of dreaming; the features in which it agrees with, and the features in which it differs from, the ordinary operations of mind. In investigating the former, in place of inquiring whether dreaming be a function or the interruption of a function, whether an active operation or a momentary passivity, it is important to consider how far analysis has established identity or similarity of action, and how far inquiry has been gravely and scientifically applied to the subject at all, either by personal self-examination, or by observation of the phenomena in others.

The gloom of the age when dreams were held to be portents, omens, predictions; when incubi and succubi had not only personality but sex, were not merely sprites, but seducers, still surrounds and overshadows us; or, in the reaction from such credulity, dreams are stigmatized as vain, idle, and as undeserving of attention. It is inculcated as a moral duty, as a protest against superstition, to disregard dreams; and the injunction is so faithfully obeyed that the mentalization of one-third of our life is, in the orthodox fear lest it should be accepted as a revelation, shut out from its legitimate place in the history of our consciousness. Yet grave, and good, and great men have, unguided by a theory, estimated the matter differently. Bossuet enters upon the subject in his *Knowledge of God and Ourselves*, and draws the conclusion that during sleep the brain is abandoned to itself, that there is no attention, inasmuch as the waking state consists precisely in the attention which renders the spirit master of itself. President Edwards has remarked that we may profitably notice our dreams in order to ascertain from them our predominant inclinations. Dugald Stewart has recorded, that an essay which he wrote in youth upon Dreams led him to those more extended researches which ended in the formation of a complete system of mental philosophy. Müller, Goethe, and our distinguished countryman, Sir William Hamilton, "three mighties," subjected their sleeping thoughts to an experimental inquiry. It is the practice to regard dreaming as something apart from our inner-life, as exuviæ to be cast off; a moral fecula which is to be expurgated. This superficial and supercilious course has led at once to ignorance of the nature of the process, misconception of its relations with more familiar mental states, and to scepticism or error as to the character of thoughts and feelings and supposed premonitions, which startle by their occurrence at times and under circumstances when their origin, connexion, or meaning cannot be detected. An illustration of this is afforded by the convictions of men of reflection and genius, that thoughts or pictures which recur by no act or will or known law of association are impressions received in a former state of existence; whereas these penumbra, these moments of sudden and brief revelations from unfathomed depths of memory, when impressions, or a series of them, originally received with faint attention, flash up in brilliant and sharp outline and distinctness, are nothing more than the recollections of dreams unnoticed at the time, or occurring in early childhood.

But when the impressions received in sleep are connected with their antecedents and subsequents, a ready solution may be found for their most grotesque aspects; they are denuded of marvellousness, and decomposable into their constituent elements. Disorder is conspicuous because arrangement has not been attempted. A

theosoph set an example in this. Bossuet saw that in dreaming of centaurs and chimeras it was absolutely necessary that the ideas of which these composite phantasms were formed, must have been recollected, compared, and then associated. It is worthy of note that when these creations of fancy were produced objectively as a revelation, a dream became a part of the superstitious, if not of the religious belief of antiquity.

That the materials of which the fabric of dreams is composed are generally recent and ordinary impressions fantastically arranged, is shown in a narrative by Dr. M'Nish :—

"I dreamed," he writes, "that I walked upon the Great Canal in the neighbourhood of Glasgow. On the opposite side stood the splendid portico of the Royal Exchange, actually some miles distant. A gentleman, whom I knew, was standing upon one of the steps, and we spoke to each other. I lifted a large stone and poised it in my hand, when he said he was sure I could not throw it to a certain spot which he pointed out. I made the attempt, and fell short of the mark. At this moment a well-known friend came up, whom I knew to excel at *putting* the stone ; but strange to say he had lost both his legs, and walked upon wooden substitutes. This struck me as exceedingly curious; for my impression was that he had only lost one leg and had but a single wooden one. At my desire he took up the stone, and without difficulty threw it beyond the point indicated by the gentleman upon the opposite side of the canal."

The elements of this patchwork are to be found in a walk upon the previous day along the canal with a friend ; a remark, on passing the Exchange, as to the improved effect which might have been secured by the selection of an elevated site ; the passing an excavation where a workman had a few days before been so crushed that both his legs had required amputation ; the recollection of a joke as to removing the leg of the friend who was the stone-throwing athlete.

The exercise of all the faculties, and in full energy, or endowed with new and greater and creative powers in the act of dreaming, might be established by citing great efforts of minds possessing and recognised by the age in which they lived as possessing, genius or understanding. Franklin avows that he was many times instructed and guided during and by dreams as to the conduct of the grave affairs which occupied his waking attention. Condillac while engaged in study continued his labours during sleep, and found, on awakening, that he had completed the process of reasoning in dream. Mathematicians, such as Cardan and Condorcet, have been able to resolve problems during sleep which had defied their investigations while the intellect was in the exercise of its waking powers, a result demonstrating the absurdity of the supposition that abstract ideas find no admission into dream.

Voltaire wrote a duplicate of the first canto of the *Henriade*, the *Divina Commedia* is said to have been inspired, and Coleridge's *Kubla Khan* was unquestionably composed during dream; and Tartini's *Sonate du Diable* is a plagiarism from a violin played by a Dream-Devil. These achievements embrace the application of the mind to some of the most difficult and elevated processes in which it can engage; but they are regarded as marvellous, less on account of their intrinsic character than of the rarity of such observations, and of the eminence of their authors. One elegant and penetrating thinker has attributed the fatigue and lassitude often experienced on awakening, to mental labour or suffering during sleep.* "It is possible," theorizes Dr. Beattie, "that this temporary suspension of our faculties may make the soul act more vigorously at other times."† But results similar in kind have occurred accidentally; and in some cases have been obtained by an act of volition in minds of ordinary calibre and capacity; and might, perhaps, be recorded of all, were due attention directed to self-examination. A more simple mode of demonstration is to subject a common dream to analysis. The sleep reverie of one who, in a sense, passed her life in a dream, was persecuted for a dream, and died a martyr for and in a dream, may be selected.

"I had a dream," says Madame de la Mothe Guyon, "which left a sweet impression on my mind. I seemed to see the wide ocean spread out before me. Many were its shoals and breakers, and its stormy waters roared. In the midst there arose an island, lofty and difficult of access where it touched the water, but in the interior where it rose again into a lofty summit, it was full of beauty. To this I was in some way mysteriously conveyed. They said it was called Lebanon; forests of cedars and all beautiful trees grew there. In the wood there were lodges, where those might enter who chose; and couches of repose were spread for them. Here in this place of Divine beauty all things were changed from what we see them in the natural world. All was full of purity, innocence, truth. The birds sang and sported among the branches, without fear that insidious foes would watch and destroy them. The lamb and the wolf were there together in peace; so that I was reminded of that beautiful prophecy of Isaiah, 'The wolf shall dwell with the lamb, and the leopard shall lie down with the kid; and they shall not hurt nor destroy in all my holy mountain.' As I thus contemplated, who should appear but that beloved one, the spouse of holy souls, the Saviour of men? He condescended to come near me, to take my hand and to speak to me. When we had looked round upon this Divine work, this new Paradise, He directed my attention to the wide waters which surrounded us, to its rocks and foam-

* Dr. Addington Symonds. *Sleep and Dreams*, 1851, p. 21.
† *Elements of Moral Science*, vol. i. p. 90.

ing breakers, and pointed out to me here and there one who was struggling onward, with more or less courage and hope, to this island and mountain of God. Some appeared to be entirely overwhelmed by the waves, but not yet wholly gone, and the Saviour directed that such in particular should receive from me whatever sympathy and aid I could give them. The sweet impression which this dream left upon my spirit continued many days."*

The congruity and continuity of this dream, and its resemblance to those moral apologues with which every nursery is familiar, at once arrest attention. It occurred before the seer attained that climax of quietism which resisted and successfully excluded the suggestions of the external senses and intellectual powers, and kept consciousness concentrated in the inner life and on the being of God. In the first place, this creation is not merely in harmony with the pietist's character and habitual thoughts, but it is a sequence of these—it imparts present gratification, it consoled her spirit for many days. She saw beautiful trees, she heard the song of birds. The spectacle of "forests of cedars" illustrates a total impression of which we are conscious, made up of an infinitude of small impressions of which we are not conscious. She had before her ideas of geographical relation in the ocean and the island, of motion in the foaming waves, of size and colour. She experienced emotions of beauty, and purity, and innocence, and sympathy, and wonder. She compared the wolf and the lamb—the struggles of the different swimmers in their agony. She drew inferences as to the absence of fear in the birds, as to the abrogation of the natural instincts of the wolf and the lamb. She manifests the intense and never-dying faith of her nature, and the realization of the very object of her daily devotion and abstraction, of seeing and being with God.

From another and still higher source it may be learned that wit and mirth may cheer the same condition, and account for the laughter which is sometimes so hearty as to rouse the sleeper. Sir T. Browne records :—" I am no way facetious, nor disposed for the mirth and gallantry of company; yet in one dream I can compose a comedy, behold the action, apprehend the jests, and laugh myself awake at the conceits thereof."†

But if the continuity advocated be demonstrable, and if a dream be merely a recollection, or a feeling under new and unusual circumstances—modified, weakened, distorted, in proportion to the force or nature of these circumstances—and distinct or faint as the mind passes through the stages of drowsiness, sleep, stupor,

---

* Upham's *Life and Religious Opinions and Experience of Mad. de la Mothe Guyon*, p. 215. 1858.

† *Religio Medici.*

re-awakening—there must arise peculiarities which, although they do not separate, may distinguish perceptions in the sleeping from those in the waking mind. It has been conceived that sleep generally takes place without dreams, and that the more normal and profound and restorative it is, the more complete and perfect is the temporary annihilation of consciousness. It is true that in the majority of persons no trace is left, perhaps because no trace is sought for, of the visions of the night. The hours of slumber appear a blank. But it is equally true that in those who have turned their attention to their dreams, who have desired, in anticipation, to penetrate into their sleeping cogitations, and have afterwards taxed their recollection, not merely fragments, but connected series are recoverable. It would appear that attention may be trained during waking to exercise its functions during sleep. Further, dreams are vivid, and impart permanent impressions in proportion to the acuteness and activity of the specific faculties, upon the operation of which they depend, or of the general mental excitement at the time. Mental tonicity, strain, and activity may postpone sleep, but are carried into it when it occurs. Great and intense thinkers are great and vivid dreamers. It has been speculatively advanced that were men to do in sleep what they dream, they would require to put on a strait-jacket in place of a night-cap before going to bed. This was written of fervid men and of frightful visitations. But the truth is, that the greater number are like the everyday thoughts of commonplace men, tame, insipid, fruitless.

Dreams are fugacious because they are feeble impressions. It is the law of consciousness to forget, the exception to remember both during sleep and complete watchfulness. Let any one attempt to recal the thoughts or emotions of a single day or hour in the order of succession, or by any process whatever, and it will soon be found that the vast majority of the impressions, vivid and interesting though they may have been, are swept away without a trace; that there occur vast gaps, during which the mind does not seem to have lived; and that the remainder are presented in unintelligible and inextricable confusion. There are states and periods in ordinary life when there are no subjective impressions, no changes in consciousness to mark the progress of time, or to be treasured up by memory; and there may be such during sleep. The laws of latent consciousness obtain, without doubt, in sleep, and may satisfactorily explain the waking forgetfulness. The more profound the sleep—in other words, the less the activity of the mind—the more entire will be the modification of consciousness, the fewer memorials can remain of the condition.

It has been noted, even by Sir W. Hamilton, that on being suddenly roused from sleep there is invariably experienced a feeling of surprise, and that under such circumstances a dream, the dream before the mind, can invariably be recalled. But the inevitable inference has not been drawn that this fact involves the necessity of there being a dream invariably before the mind. Surprise cannot create the dream, because surprise is the feeling of the awakened or awakening attention; but the forcible and alarming interruption to the continuity of the mental operations facilitates or insures the distinct recollection, exactly upon the same principle that waking thoughts are rendered indelible by their association with powerful and, above all, with painful impressions.

The rapidity with which dreams are supposed to rush through the mind, has been commented upon by one psychologist in order to establish the identity of this condition with mental alienation; by another apparently to draw a distinction between the rate of thinking in sleep and in vigilation; and by a third to show that there is no appreciation of time in sleep. But is the prodigiously long and varied procession of images presented in dreams so incomparably swift in their transit? It is impossible to watch or follow the speed of thought—and all estimates must be received rather as guesses than approximations—but many records prove that in moments of great excitement and horror, under the apprehension and feeling of immediate dissolution, there arises, instantaneously, a retrospective panoramic thought, during which the events of a lifetime may be compressed, but delineated with appalling fidelity and precision in a single moment. The disregard of the measure of time, of the epochs of history, even in our own clearest daylight thoughts, is often most reckless and capricious. We career over the events of a long and a busy life; we leap over our prescribed seven ages; we transpose eræ and unite distant periods, in a single thought. No speech, no copyist, no act can keep pace with the course of the most laggard imagination, even when in lengthened dulness long drawn out. The succession of these mental states may indeed suggest the idea of time and of its divisions, and may be the measure of passing time; but not one in a thousand individuals applies, or can apply, such a standard, and it may be safely affirmed that not one applies it in retrospect. All use the objective method—the time-honoured eight-day clock. There is a dream described by a physician in which he conceived that he had been transformed into a monolith, which stood stately and solitarily in the Sahara, and so stood for ages, till generation after generation wasted and melted away around. Although unconscious of having organs of sense, this mass of granite saw the mountains growing bald with age, the forests drooping to decay, and the moss and ivy creeping around its crumbling base. These progressive stages of decay involve the notion of time, and of time marked by natural boundaries and signs.

An experience of Lavalette while in prison is supposed, erroneously, to exemplify the velocity of thought during sleep :—

"One night while I was asleep, the clock of the Palais de Justice struck twelve and awoke me. I heard the gate open to relieve the sentry, but I fell asleep again immediately. In this sleep I dreamt I was standing in the Rue St. Honoré. A melancholy darkness spread around me; all was still: nevertheless, a slow and uncertain sound soon arose. All of a sudden, I perceived at the bottom of the street, and advancing towards me, a troop of cavalry, the men and horses, however, all flayed. The men held torches in their hands, the red flames of which illuminated faces without skin and bloody muscles. Their hollow eyes rolled fearfully in their sockets, their mouths opened from ear to ear, and helmets of hanging flesh covered their hideous heads. The horses dragged along their own skins in the kennels, which overflowed with blood on all sides. Pale and dishevelled women appeared and disappeared at the windows in dismal silence ; low inarticulate groans filled the air, and I remained in the street alone petrified with horror, and deprived of strength sufficient to seek my safety in flight. This horrible troop continued passing along rapidly in a gallop and casting frightful looks upon me. Their march continued, I thought, for five hours, and they were followed by an immense number of artillery waggons full of bleeding corpses, whose limbs still quivered; a disgusting smell of blood and bitumen almost choked me. At length the iron gates of the prison shutting with great force awoke me again. I made my repeater strike: it was no more than midnight; so that the horrible phantasmagoria had lasted no more than two or three minutes—that is to say, the time necessary for relieving the sentry and shutting the gate. The cold was severe and the watchword short. The next day the turnkey confirmed my calculations. I nevertheless do not remember one single event in my life, the duration of which I have been able more exactly to calculate, of which the details are deeper engraven on my memory, and of which I preserve a more perfect consciousness." *

There is no proof of great rapidity here; but there is proof of the conception of time, although a miscalculation of time—M. Lavalette did not know that in place of a succession of objects, he saw his march of Death as one picture, and as he might have seen the massacre of the Abbaye, or any sanguinary scene during the many reigns of terror which have decimated Paris, at a single glance. Nor did he know that under the pressure of fear or pain time is measured by agony, and appears ages.

If we gaze upon a populous country from a height, and

---

* *Biographical Sketch of Lavalette,* which originally appeared in the *Revue de Paris.*

attempt to number or realize the impressions received, the new combinations formed, the sentiments suggested, we shall feel bewildered and defeated by their multiplicity. It has been ascertained that it is possible to pronounce about 2000 letters in a minute. An actor can commit 800 lines permanently to memory in a few hours; and an accountant sums long columns at a glance, which must, however, include every figure, its value and relation. These processes all necessitate countless mental acts in a brief space of time.

There is abundant evidence that Memory is not only faithful but tenacious and far-reaching; embracing the earliest periods of life, and the nooks, and corners, and caverns of life during dream, but that, occasionally, it would appear to display transcendental powers. "There are men," says Dr. Beattie, "who observe that one particular dream frequently returns upon them. Socrates, in the 'Phædo of Plato,' tells his friend that he had all his life been haunted with a vision of this kind, in which some one seemed to say to him that he ought to study music."* We know a medical man whose officious fancy reproduced the same dream for six months. M. Maury recounts that he dreamed eight times in one month of the same subject. Visions, too, which pass away with the slumber which they have irradiated, return when the senses again lapse into hebetude. It appears that facts or trains of reasoning which have been lost sight of, or could not be summoned up when awake, have been regained in all their original force and freshness during dream. The following example is selected from the dry dull realities of every-day life. "A distinguished lawyer of the last age had been consulted respecting a case of great importance and much difficulty; and he had been studying it with intense anxiety and attention. After several days had been occupied in this manner, he was observed by his wife to rise from his bed in the night, and go to a writing-desk which stood in the bed-room. He then sat down and wrote a long paper, which he put carefully by in the desk, and returned to bed. The following morning he told his wife that he had had a most interesting dream; that he had dreamt of delivering a clear and luminous opinion respecting a case which had exceedingly perplexed him, and that he would give anything to recover the train of thought which had passed before him in his dream. She then directed him to the writing-desk, where he found the opinion clearly and fully written out; and it was afterwards found to be perfectly correct."†

In waking moments memory cannot be compelled to bring

---

* Dr. Beattie's Papers on Dreams. *Mirror*, p. 85, vol. ii.

† Abercrombie, *Intellectual Powers*. Shepherd, *On Dreams*.

before us even recent impressions. It is certain that the dreams which immediately precede waking, when the senses and attention are partially active, when the mind is sometimes awoke by its own acts, are most distinct and easily remembered. The promptitude and fidelity of this power appears to depend, in great measure, upon the clearness of the image formerly before the mind, the amount and duration of the attention bestowed upon it; in other words, upon the efficient operation of consciousness, and upon the number and vividness of the impressions with which it is associated. A poet has theorized that were there no night, no absence of the usual provocatives to attention, there would be no sleep. Now in the sleep of the healthy the senses are so far inactive, the capacity for observation of internal impressions is diminished, in consequence of the withdrawal of the stimulus of visual, auditory, and other impressions; and consciousness being simply the mind presented to the contemplation of mind, is in the same way and to the same extent less clear and comprehensive, and memory, or recalled consciousness, is correspondingly influenced. Except, when accidentally, sounds or other sensations reach the sleeper, all these phenomena are literally subjective; and if it be considered how few, even of the educated, possess the power of taking cognizance of mental states, of what is not addressed to the senses, another explanation is presented of the comparative unfrequency of recollected dreams. But, moreover, as memory depends upon association, the absence of external impressions, pictures, tastes, during sleep, which are the principal materials by which solitary ideas, or trains of thought, are reproduced, must render it difficult, sometimes impossible, to recover the features of a dream.

Such sensations besides, not merely stimulate attention and suggest forgotten perceptions, but they serve to correct the information supplied by each other, as well as the unprecise, immethodical, inconsequential results of the intellectual processes. That to the eye and ear we are constantly indebted for defining our inchoate conceptions, for supplying deficiencies, and indicating errors in our reasonings, the experience of all men can vouch. The evidence of the blind man as to colour, of the leprous man as to touch, the deaf man as to sound, may be consulted; and the result will afford insight into the state of that mind from which, during sleep, all these aids are in whole, or in part, withdrawn; and as to the inconclusive judgments, the improbable events, the distorted recollections, and disturbed feelings which constitute the stuff that dreams are often made of. As if in anticipation of the application of this theory Sir H. Holland has commented in his chapter as to the relations of dreaming and insanity, upon the influence of the admission of external sensations in correcting aberrant trains of thought.* The following case does not positively establish the instrumentality of blindness in inducing fatuity, or modified consciousness; but it goes far to show that the restoration of reason may be promoted by the restoration of vision :—

"A man was brought into the hospital at Montpellier suffering under double lenticular cataract and complete dementia. Couching was resorted to for both eyes: and on the tenth day after the operation the patient said, 'I can see,' these being the first sensible words he had spoken. As the sight improved the man became more manageable. He began to give some details as to the origin of his ailments, and six weeks after the date of his entrance he was discharged, fully capable of earning his own livelihood. Professor Bouisson added, in his remarks upon the case, that sensation stimulated the mind as electricity stimulated the nervous system, the patient being at the time favourably situated for such impressions."†

The richness and fulness as well as the clearness of dreams is regulated by the number of faculties, active and acute. The waking up of one sense or power, while the others sleep, may add sounds, or sapors, or continuous events, as the case may be. When age or disease destroys a sense, dreams are impoverished to the extent of its communications. The blind do not recall landscapes, nor the deaf music in their dreams. Dr. Blacklock, the poet, who became blind when an infant, believed, however, that he was endowed with a sense when asleep, of which he had no cognizance at other times, which brought him into contact with external bodies by threads passing from them to his body. The idea of contact, while it reminds us of the convictions of Cheselden's newly-couched patient, would be more correctly classed with modifications of the sense of touch.

The incoherence of a madman is, perhaps, little more than the enunciation of thoughts as they occur, without the key or explanation of the association by which they have been suggested or linked together. The tie may be lost, or forgotten, or concealed. Dreams appear in many instances as confused and chaotic, and from a similar privation. But this disruption is common to the course of thought in every mind. If we judge from spoken or written thoughts we shall judge erroneously; they have been carefully rethought, elaborated; they have been dislocated and rearranged; they are the products of different times; the factors must be sought in different conditions of mind; but if we note the wild and disjointed and incongruous succession of ideas and passions and inferences which pass through the mind, and where

* *Chapters on Mental Physiology*, p. 126.

† The *Lancet*, October 20, 1860.

the connexion being that of time, or place, or sound, or incongruity, may escape the thinker, we shall arrive at a close resemblance to the creations of dream.

It is a favourite dogma that will is dethroned, "three fourths abolished,"* during dream; that the ideas cannot be arranged or regulated, that our passions cannot be controlled, our appetites denied; that we are the thistle-down upon the stormy current of thought. If there be not a positive fallacy in the proposition, it is overstated. The will of the paralytic is as strong and tyrannical as that of the healthy man, but he cannot move his limb. The will in nightmare is exercised in order to escape danger or death; and we feel that we are powerless and horror-stricken, that we cannot escape. Volition exists in both cases, but is nullified by the condition of the body. A distinction should be drawn between will and the operation of will; and between will as exercised over or in the mental faculties, and will as exercised over the muscles. In all these cases there exist two psychical conditions, and these may be consentaneous or antagonistic. During every vivid dream, which is remembered, we are conscious of having desired to do, or of having been disappointed in attempts to do, many things; of having formed projects and executed or relinquished them. But there are certain facts which render it probable that volition may be exerted in sleep or in its connate states consciously, with a knowledge that it was a dream which was influenced, either by a determination formed while awake and recalled in sleep, or by an immediate and spontaneous mental act. Many individuals have, or can acquire, the power of awakening at a certain hour or minute. This is the sleeping will obeying the will of the waking mind, or it is the act of concentrated attention resulting in recalling a particular thought at a particular time. Dr. Reid is stated by Dugald Stewart to have succeeded in dispelling the frightful dreams by which he was annoyed. Whenever he dreamed that he was in danger on a bridge, or on the brink of a precipice, he cast himself down, and got rid of the scene and the terror, and apparently without recovering complete consciousness. Dr. Beattie adopted a similar expedient, and with similar success.

Dugald Stewart's theory of the absence of volition in sleep is overturned by Prevost's objection that "without will there is no attention, without attention there is no recollection of dreams." But we are apt to deify Will, and to regard our moral freedom as unqualified and absolute. Is it not unqualified and absolute? Let an attempt be made—that is, let us will—to expel a profound grief, an obtrusive remorse, to chain down a rebellious appetite, or to

* Alfred Maury, Membre de l'Institut. *Le Sommeil et les Rêves*, p. 337. Paris. 1861.

defy a fear, and it will be found how powerless the effort, how utterly enslaved the intellect and the desire for relief are by the emotive part of our nature. It is only in high natures that a victory is achieved, and then it is more rarely by direct than indirect obedience; more frequently, by the gradual weakening of the impulse than by its yielding to superior mental force, or conscience, or right reason. These struggles have suggested the words of Pascal:—"Il y a une guerre intestine dans l'homme entre la raison et les passions. Il pourrait jouir de quelque paix, s'il n'avait que la raison sans les passions, ou s'il n'avait que les passions sans raison." Moreover, it is only in high and highly-cultivated and self-disciplined minds, and under certain circumstances, that there is a conscious exercise of volition, a determinate purpose founded upon these convictions.

Müller explained to Goethe that he had not voluntary power over either the production of dream-images or their changes of form, and that they never presented the slightest tendency to a symmetrical and vegetative development. Goethe, on the contrary, was able to give a type for the phantasm; and then the different variations ensued, as it seemed, independently of the will, though with regularity and symmetry. "When I closed my eyes," says Goethe, "and depressed my head, I could cause the image of a flower to appear on the field of vision; this flower did not for a moment retain its first form, but unfolded itself, and developed from its interior new flowers. These were not natural flowers, but of fantastic forms. I was unable to fix any one form." * These phantasmata appeared as the observer was passing from drowsiness into sleep.

Maury advances one step farther—as he could not only evoke but detain the fantastic reverie for observation; and in testing this power he with good taste summoned the fascinating countenance of Madame La Vallière as the subject of this contemplation, but failed in distinguishing her toilette or figure.† Men, according to M'Nish, who never loved before, have conceived a deep affection to some particular woman in their dreams, which, continuing after they awoke, has actually terminated in a sincere and lasting fondness for the object of their visionary love.‡ In all of these instances the influence of will holds a prominent place.

The laws of pleasure and pain are likewise observed. Toothache is propagated to the sensorium as pain, although it may there assume the proportions of the rack or stake. Gratification is

---

* Müller. *The Physiology of the Senses, Voice, and Muscular Motion.* Translated by Dr. Baly. Vol. ii. p. 1395.

† *Le Sommeil et les Rêves*, p. 68.

‡ *Philosophy of Sleep*, p. 101.

found in the mere escape from sorrow and suffering, or the tear and wear of life, in the experience of new enjoyments, and in the contemplation of the suggestions of fancy. Men fly from reality to fantasy, and with the deliberate purpose of seeking greater pleasure in the one than in the other.* This has been found by men such as Sir T. Browne, whose mental power and scope must, while awake, have placed within attainment all that is glorious and ennobling in the higher realms of thought, and philosophy, and imagination, both in their own stores and in the accumulated wealth of the world. Opium, hachsich, wine, have been resorted to with the intention of intensifying these pleasures. They dull the external senses, the sense of the present, and either directly or by allaying suffering and tranquillizing the nervous system, they indirectly suggest agreeable dreams; they act as a moral dram. The brain, saturated with opium, is drunk, and, as has been written, is

"O'er all the ills of life victorious."

Hachsich is supposed to have a special power in castle-building, and in imparting exaggerated conceptions of past time.

This naturally leads to the consideration of those supersensuous efforts which, although calculated to excite surprise when manifested in dream, find a parallel in the experience of every reflecting or sensitive person. It is necessary, however, to exclude hallucinations, visions, the phenomena of somnambulism from this inquiry, as containing anormal, if not unhealthy, elements. It would appear that Aristotle was the first who announced that there was something more in dream than the images which appear to the mind—

"J'ai soutenu," says M. Maury, who, notwithstanding, contends that sleep may be dreamless, "des discussions et combiné des réponses pour parer redoubtable objections; je me suis conformé dans ma conduite au caractère de ceux dont j'évoquais le souvenir et que je faissais intervenir dans mon rêve; il y a plus, j'ai su des idées, des inspirations que j'avais jamais eues, eveillé; j'ai même trouvé certains chosés que j'avais vainement cherchées dans le recueillement du cabinet."†

Monboddo gives the case of the Comtesse de Laval, a woman of perfect veracity and good sense, who, when ill, spoke during sleep in a language which none of her attendants understood, and which even she was disposed to regard as gibberish. A nurse detected the dialect of Brittany. Her mistress had spent her childhood in that province, but had lost all recollection of the Breton tongue, and could not understand a word of what she

* *Brit. and For. Med. Rev.*, vol. xxiii. p. 223.

† *Le Sommeil et les Rêves*, p. 90.

said in her dreams. Her utterances applied, however, exclusively to the experience of childhood, and were infantile in structure.* Where somniloquism exists, not only have languages been spoken, of which the speaker was ignorant, as in the preceding case, and that detailed by Coleridge, but conversations have been carried on displaying an acuteness of intellect, an extent of information, and an eloquence and variety of speech, never displayed when awake.

All these examples have been observed during indisposition, or under circumstances productive of disturbance and excitement in the system. The brain has been roused to greater activity by galvanism applied during amaurosis. Fever has acted in the same way; and one individual who was never conscious of dreaming before, had after the attack nightly visitations.† But were the physiological side of the matter ignored, the incessant activity of certain powers, that of the memory of facts, for example, directed to a particular subject, has the effect of calling up long-lost trains of thought in dream, as well as during the effort. Not only does order sometimes, during sleep, come out of a chaos of confused and apparently irreconcilable and unmanageable materials, collected while awake, but some of the marvels and supernatural elements of dreaming are explicable on this hypothesis.‡ The following anecdote is illustrative of this point:—

"Mr. R., a gentleman of landed property, was prosecuted for a large sum of money, the accumulated arrears of tiend or tithe, for which he was said to be indebted to a noble family, the titulars or lay impropriators. Mr. R. was strongly impressed with the belief that his father had, by a form of process peculiar to the law of Scotland, purchased these lands from the titular, and therefore that the present prosecution was groundless. But after an industrious search among his father's papers, an investigation of the public records, and a careful inquiry among all persons who had transacted law business with his father, no evidence could be recovered to support his defence. The period was now at hand when he conceived the loss of his law-suit to be inevitable; and he had formed the determination to ride to Edinburgh next day, and make the best bargain he could in the way of compromise. He went to bed with this resolution, and with all the circumstances of the case floating upon his mind, had a dream to the following purpose. His father, who had been many years dead, appeared to him, he thought, and asked him why he was disturbed in his mind. Mr. R. thought that he informed his father of the cause of his distress, adding, that the payment of the sum of money was the more unpleasant to him because he had a strong consciousness that it

---

* *Ancient Metaphysics.*

† Locke. *Essay.* Book II. chap. I.

‡ *Mental Physiology.*

was not due, though he was unable to recover any evidence in support of his belief. 'You are right, my son,' replied his father. 'I did acquire these tiends, for payment of which you are now prosecuted. The papers relating to the transaction are in the hands of Mr. ——, a writer (attorney), who is now retired from professional business, and resides at Inveresk, near Edinburgh. He was a person whom I employed on that occasion for a particular reason, but who never on any other occasion transacted business on my account. It is very possible,' pursued the interlocutor, 'that Mr. —— may have forgotten a matter which is now of very old date; but you may call it to his recollection by this token—that when I came to pay his account, there was difficulty in getting change for a Portugal piece of gold; and we were forced to drink out the balance at a tavern.' Mr. R. awoke in the morning with all the words of the vision imprinted upon his mind, and thought it worth while to walk across the country to Inveresk, instead of going straight to Edinburgh. When he came there, he waited on the gentleman mentioned in the dream, a very old man. Without saying anything of the vision, he inquired whether he remembered having conducted such a matter for his deceased father. The old gentleman could not at first bring the circumstance to his recollection; but on mention of the Portugal piece of gold, the whole returned upon his memory. He made an immediate search for the papers, and recovered them; so that Mr. R. carried into Edinburgh the documents necessary to gain the cause which he was on the verge of losing."*

Here the labours and efforts of recollection culminated in the discovery of the pivot incident; and it is instructive that the memory of the aged lawyer was roused, and recovered the lost train of thought by the same means.

Jerome Cardan, who afterwards composed books while asleep, saw, immediately after awakening, figures which followed each other in long procession, were of many kinds, houses, castles, animals, knights on horseback, plants, trees, musical instruments, and wild shapes, that represented nothing he had seen before. He had pleasure in the spectacle, and made a secret of it.

There are many instances of daymare, in which oppressed respiration, appalling visions, extreme dread, the inability to move or speak, usurp the healthy consciousness; and a literary man, of comprehensive intellect, as well as fine imagination, has told us, with a shudder, of awakening in nightmare, which he could not cast off for hours, and during which there was added to the characteristic panic,—the struggle of volition to acquire supremary over the recusant, or lead-like, or dead inertia, which seemed to weigh him down, and the agonizing conviction that he never would get out of the deathlike thraldom. Much attention

---

* Sir W. Scott's notes to the *Antiquary*.

has been paid to the state of the mind immediately previous to sleeping. The moment of awakening is more pregnant with results. Dreaming has scarcely ceased, and the excitement of the emotions which it produces is still present; we see, but do not appreciate relations. There is a certain control over the muscular system, but the interpretations of the impressions, and the impulse to move, may be determined by what has taken place in dreams. It is only known to us as bloodstained. A man awaking at midnight, beholds a frightful phantom; fear and the obscurity of the night prevent him from observing objects distinctly. In a tremulous tone he called out, Who goes there? The apparition approached him, but he received no answer. Much frightened, he seized a hatchet, and in a moment, and without reflection, he felled the phantom with a single blow. His own groan, the fall of the object of his terror, restored him to a clear perception of surrounding objects, and to the knowledge that he had killed his wife, who slept in the same room.*

But this condition, if looked for, may often be discovered among the confined insane. One case fell under our own care, where, during the intervals between paroxysms of mania, a female slept much, and on being roused, passed a quarter of an hour in a waking dream, during which she saw, but did not recognise those around; spoke incoherently in reply; tied her cap and arranged her dress. This period was selected for removing a collection of pins which she was in the habit of accumulating under the tongue and between the cheeks and gums.

Identical with the harbingers, or hypnogogic hallucinations as they are called, and with the sequelæ of sleep, are the phantasmata of the German physiologists. These are visible to certain gifted or perverted minds. Many facts render it probable that by concentrated attention such images may be called up by all. It has been calculated that one or two per cent. of healthy individuals become cognizant of such impressions. To females, but especially to susceptible children, they are most frequent visitants. When sleep is coming on, or departing, landscapes, faces, and other objects float around and are traced upon the walls or curtains. If seen when the eyes are shut, they may be reflected in a fashion upon a neighbouring object by opening them. They may be seen in whatever direction the head is turned, but they do not move with the eyes. The theory propounded for the explanation of these appearances, and of the true hallucinations of the unsound mind, is that the idea in the sensorium excites the active state of particles of the retina or optic nerve. But that the retina is not necessarily implicated in the production of these,

* Hoffbauer.

is demonstrated by the appearance of luminous figures before the orbit, subsequently to the extirpation of the eye; and in cases where complete atrophy of the optic nerve has been found after death. Sometimes these objects are exact transcripts of scenes, carpets, furniture formerly seen, and sometimes they are new creations. They remain sufficiently long for careful scrutiny; sometimes after the modified consciousness in which they originated has given place to normal perception, and are generally luminous, highly colorated or variegated.

It has been observed that such individuals as most frequently experience hypnogogic hallucinations, or draw erroneous conclusions from actual perceptions received when falling asleep, are of an easily excitable constitution, and are generally predisposed to hypertrophy, pericarditis, and cerebral affections. Of this class were Burdach, Purkinje, Müller. From the nature of many of their revelations it may legitimately be inferred that they were unhealthy as well as hyperæsthetic men. They were lay figures, subjects of experiments to themselves. The prodigality and detail and refinement of their experiences, however, suggest that these may differ from those of other men. M. Maury finding "mes hallucinations sont plus nombreuses et surtout plus vives, quand j'éprouve, à qui est fréquent chez moi, une disposition à la congestion cerebrale," was naturally led to systematize his dreaming. While Swedenborg journalized his visits to other worlds, his transcendental impurities, M. Maury kept what may be styled a noctuary of dreaming, extending over years, including accidental impressions during sleep, as well as cunningly devised experimental researches. Whenever he fell asleep he was awakened suddenly, and at all hours, and the dreams at which he had arrived carefully noted, compared with external circumstances, and with each other. He had likewise a fortunate facility in recalling with distinctness whatever had occupied his mind. He quaintly says that it was his office to depict the mind in dishabille, and that he was necessitated to reveal many errors and nervous defects.

When he pertinaciously devoted himself in the evening to severe work, hallucinations invariably appeared. Having for some consecutive days attempted to translate a long and difficult passage in Greek, he saw, on lying down in bed, the characters so multiplied, and careering so swiftly before his eyes, that he started up in absolute terror. There is mentioned in Combe's *System of Phrenology* a boy who was made ill by application to his tasks. "He slept ill, for the curtains and counterpane and pillow, which were white, were all written over with Greek grammar." "But you should have closed your eyes," advised the teacher. "That would have done no good," said the sufferer,

"for I still saw the passages of the Greek grammar after my eyes were shut."*

Coffee and champagne provoked the visions in M. Maury; and the apparitions of these hallucinations awoke him. Dropping repeatedly but momentarily asleep while reading Hommaire de Hell's *Voyage*, whenever he closed his eyes, the figure of a Capuchin monk restored him to consciousness. He has often satisfied himself of the identity of these images with the figures which are presented in his true, or profound dreams. He relates that, feeling hunger in consequence of a restricted diet, he saw in the intermediate state between sleeping and waking, a mess presented by a hand holding a fork. On sleeping more soundly, the temptation was continued by finding himself seated at a well-served table, and by hearing the sounds of the forks of the guests. He has satisfied himself of the sensorial origin of the hypnogogic hallucinations of sight. For example, when suffering under what he conceives to have been congestion of the retina, he saw coloured flies and luminous circles designed on the eyelid, and when assured of the invasion of sleep by the appearance of fantastic images, he has often found that the brilliant objects, attributable to the state of the nerves, were altering themselves in some sort under the eye of imagination, and were transformed into a figure of grotesque but luminous aspect. He conceived it possible to follow for a few seconds the metamorphoses effected by his mind upon this primitive nervous impression. He further believes that the coloration, the lustre, and the paleness of such images when the phenomena begin to fade, demonstrate that they originate in excitation of the retina, induced or kept up by irritation or congestion of the brain. According to Purkinje, these images are changed whenever the muscles compress the globe of the eye, and, according to Muller, they may be dispelled by the slightest movement of the organ. Such visual impressions may acquire intensity, so as to pass into true dream, or, into visions seen when the eyes are open in darkness. The same obtains as to hearing. Voices or short words are heard at a distance, or deep in the interior of the head. When on his way to Strasbourg, M. Maury fell asleep on the top of the diligence, fatigued by the night journey. He speedily heard German spoken, although still at a distance from Alsace. There was no German passenger. After a brief struggle to dispel the fancy, he again fell asleep, and the words which then reached him were a mixture of Dutch and German. Suffering under tinnitus aurium, the expectation of listening to a new language seems to have evoked it in the uneasy slumber which fatigue produced. A

* P. 213, Vol. ii. Fifth edition.

vision of roast pork and sausages evolved in their train the distribution of these comestibles to the National Guards charged with the defence of the Luxembourg in June, 1848. There was then suggested the grave apprehensions of that crisis, then a visit to Barcelona, and while there indulgence in cookery with rancid oil. The unsavoury sauce persecuted him in sleep once and again.

That we rarely dream of the pleasures of the table, and of the aroma of the viands, must have been alleged by an ascetic, or by one who, like Wordsworth, smelt the breath of flowers but once. We believe that the comparative rarity of the co-operation of the senses of smell and taste in dreams, and the order in which the other senses contribute to the composite effect is strongly corroborative of the propositions advocated. Rabelais jests of a people who heard with their eyes and understood with their elbows. A vast number of men think and recollect by their eyes. Picture-ideas are the only ideas of which they have any conception.*

A peasant found himself ever in an atmosphere of sickening stench when on the verge of slumber, and regarded it as the natural effluvium of the devil, as, whenever he smelt it, he saw hideous figures. These errors must sometimes be contemporaneous with irritation of the mucous membrane of the nose or stomach, although they may likewise be connected with excitement of the nerves of taste and smell, independently altogether of the condition of other tissues. Such examples, however, show how close the alliance is between dreams and hypnogogic hallucinations. There may be doubt, indeed, as to the existence of any difference between the impressions during the obscuration of consciousness and its eclipse. The latter are experiences of men of certain temperaments only. The election of the time in which they occur is altogether arbitrary, and there is no element in their nature which distinguishes them from genuine dreaming except their apparent vividness, and this depends upon the degree of activity in the mind at the time when they are perceived. But, as far as such observations are trustworthy, they indicate the uninterrupted operation of the mind, and its regulation by the same laws as when fully vigilant.

It has struck observers that there is an error in regarding sleep as a single condition. It certainly comprehends various gradations and stages of the same condition, from the flower-like sleep of the child, to the convulsive throes of the conscience-tossed; and these are, perhaps, different in nature, and are

* It has been shown by Thore and Aubanel that the waking hallucinations of taste and smell stand to those of sight and hearing as 9 to 97.

assuredly modified by internal and external circumstances. Somnambulism may be a form, but abstracted men, in whom, from engrossment with a particular train of thought, there is inattention to the reports of the senses, and whose monoideaism, as much as the fixed gaze of the eye upon an external object, predisposes the mind to sleep and dream; lethargic men in whom sensation is slow, and hebete, and inaccurate; demented men in whom sensibility is dulled or abolished; hypochondriacal men in whom it is perverted—all approach the sleeping and dreaming man. But there are cases where a species of sleep and dream spread over whole periods of life, and render it doubtful to the individual how far the external world is realized, and what belongs to the experience of a vigilant, and what to a dreaming consciousness. In the insane, and in many who, though not insane, are of unhealthy mind, such cases occur. A governess, for many years under our care, "awoke," as she herself expressed it, after a sleep of sixteen years, her absurd fancies and exquisite misery appearing to her as the faint, hazy features of a dream. A lady now alive testifies that she passes months in dream while actually engaged in the discharge of her ordinary duties. Even Fenelon appears to have entertained doubts as to the point at which Madame de Guyon's spiritual life or holy dreaming passed into ordinary mundane existence.* There are men who mingle day-dreams with realities, who see a world that we, the vigilant, cannot see; where the external senses are partially dormant, and where it becomes impossible to distinguish between a dream and a deliberate judgment, a castle built by fancy in cloudland, and the creation of a sane imagination, except by the extravagance of the latter.

"For years Hartley Coleridge seems to have lived a double life, and the faith which the child reposed in the inner world was at least as strong as that with which he regarded the outer. At a very early period of his childhood, of which he himself had a distinct though visionary remembrance, he imagined himself to foresee a time when, in a field close to the house in which he lived, a small cataract would burst forth, to which he gave the name of Jugforce. The banks of the stream thus created soon became populous—a region, a realm; and as the vision spread in ever-widening circles, it soon overflowed, as it were, the narrow spot in which it was originally generated, and Jugforce, disguised under the less familiar appellation of Ejuxria, became an island continent, with its attendant isles—a new Australia, if it were not a reflection of the old Europe projected from the clouds on some wide ocean somewhere. The history and geography of this region were at one time familiar to me, to say the least, as any other portion, I was about to say, of the habitable globe. The details

---

* Bayle, *Hist. and Crit. Dict.*, vol. ix. Butler's *Life of Fenelon.*

have gradually faded from my memory, and fitly enough, no written record remains, though an elaborate map of the country was once in existence, from which they could be recovered. Taken as a whole, the Ejuxrian world presented an analagon to the world of facts, so far as it was known to Hartley, complete in all its parts, furnishing a theatre or scene of action, with *dramatis personæ*, and suitable machinery, in which, day after day, for the space of long years, he went on evolving the complicated drama of existence." "Nothing could exceed the seriousness of his manner, and doubtless of his feelings, when saying he had received letters from, or had visited, Jugforce. He was, I am persuaded, *utterly unconscious of invention*. I have reason to believe that he continued the habit mentally from time to time after he left school, and, of course, had no longer a confidant. A certain infirmity of will, the specific evil of his life, had already shown itself. His sensibility was intense, and he had not wherewithal to control it. He could not open a letter without trembling."*

This appears the reproduction in day-dream of his father's Pantisocratical Society on the banks of the Ohio.

Instances of insanity following and being induced by dream are recorded. A case came under our own observation where panphobia began in and by a dream; where the sufferer fell asleep tranquil and rational, and in good health; where the terror of impending death at the hands of the executioner destroyed reason and self-possession, and where the victim awoke a terror-stricken maniac. This is an instance of the propagation and perpetuation of the sentiment excited by dream as an element of the after-life. There are rare cases where, like Eugene Aram, the

> "Methought last night I wrought
> A murder in a dream"

runs along the stream of life and is ultimately realized, although not perhaps foreshadowed. A man was executed for murder in Jedburgh, in 1823. "Some years before his crime he dreamed that he had committed murder, and was greatly impressed with the idea. He frequently spoke of it, and referred to it as something ominous, till at last it was realized."†

In all these aspects sleep is viewed not only as one simple condition but as indivisible; but, in order to obtain a comprehensive conception of its modifications and its relations with the exercise of the mental faculties, it would be expedient to recognise, 1. Sleep with agitating dreams; 2, somniloquism; 3, sleep with nightmare; 4, somnambulism; 5, artificial somnambulism; 6, day-dreaming; 7, double consciousness; 8, trance; 9, hypnotism;

* "Memoirs of Hartley Coleridge," quoted in *Edinburgh Review*, July, 1851.
† Combe's *System of Phrenology*. Fifth edition, vol. ii. p. 215.

10, abstraction; 11, dementation, as belonging to the same category in which the ordinary mental operations are shadowed forth; where memory supplies materials; simple suggestion associates; judgment compares; reason enters upon sequences and sometimes complicated reasonings, and spiritualized conceptions; and where joy and sorrow arise according to the waking temperament and tendencies, and in exact proportion to the intensity and exercise of these.

There is a fallacy in arguing the identity of dreaming and insanity because certain of the phenomena in each are assimilated. Dr. Rush is adduced as holding that a dream may be considered as a transient paroxysm of delirium; and delirium as a permanent dream. Dr. Abercrombie entertained a similar opinion. Sir H. Holland says, more cautiously and philosophically—"A dream put into action," as we have seen dreams are put into action, "might become madness in one or other of its most frequent forms, and conversely insanity may often with fitness be called a waking and active dream."* The psychical phenomena in both dreaming and insanity are the results of the mind acting under unusual if not anormal physical conditions; and where dreaming depends upon diseased structure it may merge into some form of derangement. But, in the first place, the physical condition, whatever that may proximately be, is widely different in the two states; in the second place, the supposed similarity can only be traced where hallucinations are among the symptoms of insanity; in the third place, the duration, the character, the effects of dreaming are, to a considerable extent, under the control of the healthy will; and in the fourth place, the broad, the irreconcilable difference between the dreams of the sane and of the insane establish a distinction at the very point where it might have been expected that the two states might approximate. The dreams of the madman are as incoherent and absurd as his imaginings, they are a confused or faint duplicate of his delusions and extravagances, or they afford, or should afford, a diagnostic mark of his alienation. Some years since, 1842, a series of inquiries were made into the amount of sleep and the mental condition of the insane during the night, and the following is an epitome of the observations and results. The obstacles to such an inquiry were very great, but in many cases it was possible to follow the patient into his most secret thoughts. A record was kept of all remarkable dreams, phantasies, and visions, which made so deep an impression as to influence the conduct of the individual, which excited some powerful emotion at the time, or were afterwards communicated. Certain individuals were selected who were of

* *Chapters on Mental Psychology,* p. 110.

marked character, whose habitual trains of thought were well known, and whose disclosures were spontaneously made and could be accepted as trustworthy. The scheme established, so far as it went, the identity of the dream with the delusion, and showed that the current of morbid thought flowed on uninterruptedly through the agitation and vivid impressions of the day and the quiet and repose of the night. In some instances it appeared that previous acts and feelings entered into and coloured and directed dream. Thus immediately after a festive meeting, the visions of one of the party were found to contain a picture of glittering and gorgeous dresses, and of another to display a dance performed by the wives of the Goths and Vandals in St. Paul's Cathedral. Much more frequently the dream, regarded as a reality, moulded and modified the conception and delusion of the waking state. Thus one man was persuaded that he was destroyed by magnetism, silent combustion, and complained to the authorities that attempts were made upon his life; a second dreamed that he was possessed of corn and wine and oil, and distributed them next day; a third that he saw the books of the nation in the hands of the Lord Chancellor and that a long black stroke was drawn across the national debt, and in the morning he announced that he was about to pay it. It would be as difficult to convey any adequate notion of the extravagance, the grotesqueness, and sometimes the splendour of these reveries, as to follow the unsound mind through all its waywardness and wanderings; but it was consolatory to discover that the prevailing characteristics were pleasure and happiness. Of about seventy dreams detailed with great minuteness, four owed either their origin or their predominating features to fear; seventeen to the gratification of some sense or the realization of some hope or ambition; four sprung from reawakened affection or humanity; three were occupied with political triumphs; three were disturbed by anger; nine were tinged with the harmless superstitions of early days; five seemed to be the offspring of vanity, two of pride, nine of avarice or the desire of aggrandizement; three were busied with martial pomp and glory, and three with the more peaceful operations of the farm; and of the total number, whether pure creations of imagination or indistinct and distorted recollections of past events, not above fifteen could be regarded as giving pain or suffering and as calling for sympathy. All were, more or less, believed in.

Among the curiosities in dreaming experience, and as illustrative of the inability of the mind to interpret sensations correctly, may be enumerated the dream of Dr. Gregory, that he was ascending Mount Etna and found the heat of the ground almost intolerable, suggested by the application of a hot bottle to the feet; of Des Cartes' starting from the bite of a flea, under the conviction that he was transfixed by a sword; and of a patient to whose head a blister had been applied, that he was scalped by the North American Indians. These observations show, however, a certain analogy between the *nature* of the external impression and the conception in which it became embodied, as well as a correct localization of the irritation. Similar conclusions are warranted by the experiments of Girou de Buzareingues, who, in order to test the power of determining previously the character of the dream, left his knees bare during the night, and dreamed that he was travelling in a diligence, where the limbs invariably become cold; and leaving the back part of the head exposed, dreamed that he was present at a religious ceremony in the open air.

A narrative which has been produced in various works upon this subject as a marvel—and many of these narratives have become stereotyped—may here be introduced as illustrating the intimate connexion between present impressions and dreams. Diluted, perhaps filtered, it is extracted from *Curiosities of Medical Experience*:—

"The subject of this observation was an officer in the expedition to Louisburg, in 1758, in whom the faculties continued so alive to the slightest impressions, that his companions were in the constant habit of amusing themselves at his expense. They could produce in him any kind of dream by whispering in his ear, especially if this was done by a friend with whose voice he had become familiar. One time they conducted him through the whole progress of a quarrel which ended in a duel; and when the parties were supposed to have met, a pistol was put into his hand, which he fired and was awakened by the report. On another occasion, they found him asleep on the top of a locker in the cabin, when they made him believe that he had fallen overboard, and exhorted him to save himself by swimming. They then told him that a shark was pursuing him, and entreated him to dive for his life. He instantly did so, and with so much force as to throw himself from the locker to the floor, by which he was much bruised, and awakened of course. After the landing of the army at Louisburg, his friends found him one day asleep in his tent, and evidently much annoyed by the cannonading. They then made him believe that he was engaged, when he expressed great fear, and showed an evident disposition to run away. Against this they remonstrated, but at the same time increased his fears by imitating the groans of the wounded and the dying; and when he asked, as he often did, who was hit, they named his particular friends. At last they told him that the man next to himself in the company had fallen, when he instantly sprung from his bed, rushed out of the tent, and was roused from his danger and his dream by falling over the tent-ropes."*

The errors committed in this state may be explained in the

* *Curiosities of Medical Experience*, by J. D. Milligen, M.D., p. 301.

same way. A dream arises that we clutch a corpse that lies beside us. On resuscitation we are in the act of grasping the left arm, on which we are lying, with the right hand. The bloodvessels and nerves of the limb have been compressed in the position occupied; it is benumbed, cold from exposure, in popular phraseology *dead;* from it, at all events, no impressions are conveyed to the brain, and it is thus essentially separated from consciousness, which imperfectly intimates the separation, while the sentient hand conveys the sensation of coldness. With a view to determine the extent of the instrumentality of the senses in the production of dreams, M. Maury submitted to a series of experiments in which the external organs were provoked in a manner and by substances of which he was ignorant, while he was asleep; and the succeeding dream immediately and carefully noted. The results of some of the significative observations may be detailed.

1. His lips and nose were tickled by his coadjutor with a feather. He dreamed that he was subjected to horrible tortures, that a pitch plaster was applied to his face, which was then roughly withdrawn, denuding the lips and cheeks.

2. A pair of tweezers were struck close to his ear by scissors. He dreamed that he heard the ringing of bells, which speedily passed into the tocsin, and suggested June, 1848.

3. He was made to smell Eau de Cologne. He dreamed that he was in the shop of a perfumer, which led the fancy to the East, and to the shop of Jean Farina in Cairo!

4. He was made to feel the heat and smell of a burning match; and the wind at the time whistled through the shutters. He dreamed that he was at sea, and that the powder-room of the vessel blew up.

5. His neck was slightly pinched. He dreamed that a blister was applied; and then there arose the recollection of a physician who had treated him in youth.

6. A piece of red-hot iron was held close to his face for such a ength of time as to communicate a slight heat. He dreamed of bandits who got into houses and applied hot irons to the feet of the inhabitants, in order to extract money from them. This idea suggested that of the Duchess d'Abrantes, who he conceived had chosen him as secretary, in whose memoirs he had read of chauffeurs, or bandits who burned people.

7. The word "parafaramus" was pronounced close to his ear. He heard nothing; but on a repetition of the attempt while in bed, the word "maman" was followed only by a dream of the hum of bees. When the experiment was repeated some days subsequently, and when he was falling asleep, he dreamed of two of three words, "Azor, Castor, Léonore," which were attributed to the interlocutors in his dream. The repetition of "chandelle, haridelle" awoke him while pronouncing the words "c'est elle;" but without any recollection of the idea attached to the expression.

8. A drop of water falling on the brow suggested a dream of Italy, great thirst, and a draught of Orvietto.

9. A light surrounded by a red paper was repeatedly passed before his eyes. He dreamed of a storm of lightning which reproduced a violent tempest which he had encountered between Morlaix and Havre.

It is very doubtful whether a waking mind emasculated by the deprivation or disease of several of the senses could have arrived at more precise results.

There may be added to these facts, observations collected by M. Maury in that cultivation of the dream power which he seems so assiduously to have pursued. He brings them forward as examples of false association; but they appear entitled to admission to the class of legitimate simple suggestions by means of sound, juxtaposition, or antagonism. "I have often," he says, "on awakening, recalled, and by reflection endeavoured to reconstruct the impressions which had occupied me during the night, not, be it understood, like the ancient Egyptians, in order to draw from them rules for future conduct, but in order to raise the veil which covers the mysterious origin of dream." Upon one occasion he had in sleep made a pilgrimage to Jerusalem, but could not positively say whether he was a Mussulman or a Christian. At the close of forgotten adventures he found himself in the Rue Jacob, in the shop of M. Pelletier, the chemist, who in conversation gave him a pellet of zinc, which played an important part in subsequent dreams which could not be recalled. These three ideas, these three scenes, are absolutely bound together by the three words "pélerinage, Pelletier, pellet," commencing with the same letter, and associated rhythmically. On recounting this fact to a friend he afforded a similar experience. The words Jardin, Chardin, and Janin, were so intimately connected in his imagination that he saw in sleep the Garden of Plants where he encountered Chardin, the traveller, in Persia, and who, to his astonishment presented to him Jules Janin's tale of the *Dead Ass and Headless Woman.* Another form of association is thus described: "I had thought of the word kilomètre so much that I was occupied in a dream in passing along a road and in reading the measured distances upon the milestones. Suddenly I found myself in one of those scales used by grocers, upon one of the plates of which the shopman heaped up kilos in order to determine my weight. The grocer next announced that I was not in Paris but in the island of Gilolo, of which I had scarcely ever thought before. The fancy stepping from the associations of the first to those of the last syllable brought before me the flower lobelia, General Lopez who perished in Cuba, and a party at loto, of which I was one."

It is not only "for in that sleep what dreams may come," but what dreams may come from that sleep, mingle with our thoughts, and become as part and parcel of the self-conscious mind,—motives and guides to action.

Ideas, recollections, become present to us without any or with very imperfect knowledge of when or how they originated, and there is, *à priori*, no guarantee against our using cognitions from a spurious source, of our obeying the dictates, resorting to the arguments or inferences, of a dream unconsciously. But there is evidence that in certain cases where the actor is ignorant that the impulse is the offspring of states occurring during sleep, and in certain others where there is a suspicion and shadowing that no waking moment produced the feeling, that it has acquired dominion over the faculties, or over the ordinary mode in which they are exercised, and has determined a life crisis or a continuous course of action. This involves the ethics of dreaming. Men are held to be morally responsible for the impure and uncharitable or vicious thoughts which they entertain or cherish. Crimes are often devised or committed during sleep, of the most hideous and horrible kind, and it becomes a nice question how far the design and act are the sequence or reflection of the natural character and course of thought, and how far blame may attach to a mind so imbued and predisposed. There is conscience in dream. Every one must recollect of having deplored an assault or a homicide, and of having attempted to fly from merited punishment; nor is the conviction of personality so much weakened as to alter the nature of the imaginary delinquency. To the pure all things are pure and at all times, and it is obvious that the unpolluted heart and the well-regulated reason will not overflow, except under the pressure of disease, in grossness or violence. But the dreamer may commit suicide in dream, as appears from an inquest held in London in 1842; or he may rise during sleep and consummate a foul sensual impulse which may have arisen while awake, but been matured in dream. We knew of an educated man who lived as a bachelor in a house with two female servants. Upon him one of these affiliated an illegitimate child. He pertinaciously and long denied all conscious sexual intercourse with the woman; but at last admitted the paternity on the grounds that he was a somnambulist, and had repeatedly left bed and ridden out in that state,—that he was aware of having experienced erotic tendencies towards his servant, and remembered that he dreamed of having had connexion with her upon the occasion libelled. There is abundant evidence that as, in one aspect, dreams are the shadow of the substantial realities of life, the last pulses of passion, they may as shadows of impurity and passion penetrate into the unguarded or partially guarded moral condition during sleep. Adopting this view, saints, or, in this sense, sinners, have sorrowed over and done penance for the shortcomings or the crimes of sleep. St. Anthony's temptations and struggles may have taken place in reverie or slumber, or when on the verge of either. We would not desecrate the revelations of St. Theresa by stigmatizing them all as dreams, but her own confessions appear to demonstrate that many must have been vouchsafed during her trance-sleep of a quarter of a century. She, the most practical of mystics, is, moreover, recorded by her most recent and her Protestant eulogist to have carried her devotional feelings so thoroughly into every mental state, as to have "prayed in sleep."*

A conspectus of the opinions held by those who have investigated the psychical aspect of sleep, and in a philosophical spirit, may present it in the various lights of which it is susceptible. Leibnitz argued that even when we sleep without dreaming there is always present some faint perception. Sir W. Hamilton unfortunately attempts to prove the postulate by reference to somnambulism. Kant argued that we always dream when we sleep; that those who fancy that they have not dreamt have only forgotten their dream, and that to cease to dream would be to cease to live. Marie de Biran conceives that the soul sleeps and that the senses are torpid. Geoffroy argues that the mind wakes, that certain of the senses transmit the sensations which they receive, and the mind awakes the senses by its own activity. Lelut controverts these opinions, and argues that there is neither suspension nor vigilance, but repose. Dugald Stewart, while holding uninterrupted psychical action, affirmed that the activity and volition of the spirit were suspended. Euler believed that the more the influence of the senses is suspended, the more regular and connected are our dreams. He does not carry this theory so far as Democritus, who put out his eyes in order that he might philosophize the better; or as Malebranche, who closed the shutters and excluded every ray of light, that he might think intensely; or as St. Anthony, who complained that the rising sun deprived him of the "greater interior light." Müller thought sleep the antagonism of the organic and animal functions. Burdach calls sleep the primordial state of the soul, where it finds itself when it awakes to life. Maury compares the condition of the sleeping man to that of the fœtus; and Macario, while admitting that dreams between sleeping and waking have demonstrated that the exercise of the faculties is not entirely

* "Santa Teresa." *Fraser's Magazine*, January, 1862, p. 66.

suspended, designates sleep as the negation of the integrity of the moral and intellectual life.

M. Macario* divides physiological dreams into, 1, Sensorial or intracranial, where they apparently arise from the mind itself and without the instigation of a sensation, as may be exemplified in the case of a woman hearing for three consecutive nights a voice exclaiming, "Kill thy daughter, kill thy daughter," and who immolates her child. 2, Into dream illusions where an external impression stands in the relation of cause to the mental condition which follows. A dreamer hears an explosion and immediately conceives that he is in the presence of conflicting armies; he sees the blood flow, he hears the discharge of musketry and cannon, the clash of arms, the cries of the combatants and the groans of the wounded and dying; or the sound of a bell recalls the days and joys of infancy, or the gloomy pageantry of a funeral or a religious solemnity, according to the sensibility of the individual. His second class of physiological dreams is the emotional, which are described as those in which the affections and passions predominate, and which he traces to the ganglionic system. The exile, a prey to nostalgia, dreams of his native country, his home, the objects of his love, and he hears the voices of his kindred. Love may suggest lascivious dreams; shame may present the dreamer exposed nude to the gaze of a multitude; crime may conjure up remorse and horrors; hunger may spread a table with good cheer. The third class is the psychical, or intellectual, where in sleep the sphere of intellect extends and enlarges itself in a marvellous manner; the ideas are more vivacious and lucid, the imagination is bolder, the memory more precise, the judgment more prompt and assured. It might be said, he breaks out poetically, that the spirit bursts the bonds which connect it with earth, and launches itself into the ethereal regions, into the dazzling sojourn of truth. Many successful literary and scientific efforts have been inspired by an intellectual dream. Galen acknowledged that he owed a great part of his experience to the lights received in dreams. Hermas wrote his *Pastor* to the dictation of a voice heard in sleep.

Morbid dreams are classified as, I. Prodromic, or where certain well-marked mental conditions during sleep precede and are supposed to signalize the approach and commencement of remote bodily ailments. The soil is virgin; and even where isolated observations have been made, it is not easy, in all cases, to connect the premonitory symptom. M. Teste, Minister of Justice, dreamed that he had suffered from apoplexy, and actually died of the disease in three days afterwards. A woman dreamed that she

---

* *Du Sommeil, des Rêves, et du Somnambulisme dans l'état de Santé et de Maladie.* Lyons, 1857.

addressed a man who was mute, and awoke aphonic. Conflagrations and sanguinary spectacles are said to announce attacks of hæmorrhage. The origin of deep-seated and obscure affections of the abdominal and thoracic viscera may, in the absence of other signs, be indicated by dreams. Nightmare and disturbed dreams are premonitory of nervous diseases. II. Symptomatic. The illusions of pretended sorcerers, of vampires, may fairly be identified with their dreams. Esquirol has discovered in the revelations of a sleep-talking monomaniac information which he had been foiled in obtaining during his waking moments. The dreams of the insane are generally characteristic of the nature of the aberration under which they labour; those of the lypemaniac are gloomy and frightful; of the general paralytic, gay and smiling; of the maniac, wild, disordered, pugnacious; in stupidity they are vague, obscure, and incoherent; in dementia few and fleeting. In hypochondriasis and hysteria the sleep, especially during digestion, is disturbed and painful. It is said that in persons affected with chorea the dreams chiefly refer to the sense of hearing, owing, it is imagined, to the state of the circulation or the impoverished blood circulated. III. Diseased dreams include ordinary, periodic, and epidemic nightmare; somnambulism, and various congeneric conditions. The first is characterized by visions of a frightful and painful nature, and by the feeling of fear; the second by the exercise of the muscular system, the external senses and other organs during dream, and under the direction of the mental powers then active. As instructive as to that rare phenomenon, epidemic nightmare, the following narrative may be quoted:—

"The regiment of La Tour d'Auvergne, when in garrison at Palmi, in Calabria, received orders to start at midnight, and to reach Tropea by forced marches, in order to oppose the landing of the enemy, who threatened the harbour. It was in the month of June; the troops had to traverse a distance of about forty miles; leaving at midnight, they arrived about seven o'clock in the evening, and having rested for a short time only, they suffered considerably from the heat of the sun. As the battalion was the last to arrive, they were apportioned the worst barracks; and eight hundred men were crowded into a building which, under ordinary circumstances, could not have contained above the half. They squatted upon straw laid upon the earth, without covering, and without undressing. The building was an old deserted abbey. The inhabitants of the locality warned the soldiers that they would not be able to retain even this lodging, that it was haunted nightly by spirits, and that other regiments had tried in vain to obtain rest there. We laughed at this credulity, but were much astonished when, at midnight, there issued from every corner of the ruin the most frightful cries, and to see the soldiers rush out and flee panic-struck. When questioned, they all declared that the devil inhabited the abbey, that they had seen him enter by an opening in the door of their apartment under the form of an enormous black shaggy dog, that he rushed at them, crossed their breast with the quickness of lightning, and disappeared upon the opposite side to that at which he came in. We ridiculed their terror, and endeavoured to prove that the phenomenon depended upon simple and natural causes, and was, in fact, the result of the excited imagination. We failed, however, in convincing them, and in persuading them to return to their quarters; and they passed the night upon the beach and scattered through the town. When the sergeants and oldest soldiers were interrogated upon the following day, they declared that they were inaccessible to any sort of fear, that they did not believe in ghosts or hobgoblins, and appeared persuaded that the scene in the barrack was a reality, and not to be attributed to imagination, as they were scarcely asleep when the dog rushed in, that they saw the animal distinctly, and that they felt suffocated at the moment when he jumped upon their chest. The regiment was detained in Tropea, and occupied the same building on the following night. The men had been spoken to, and were comforted and supported by the presence and the scepticism of their officers, who placed themselves in the different chambers; yet about one o'clock the same shrieks arose, and the same individuals who had been assaulted by the hell-hound on the previous night rushed in trepidation into the open air in order to escape suffocation. The watchers saw nothing but the panic."*

The surgeon of the regiment, who is the narrator, adds, that although repeatedly exposed to similar hardships, he had never witnessed his comrades so affected, and attributes the epidemic to the state of the atmosphere and other very obvious circumstances. Every event is explicable save the simultaneous appearance of the same spectre at the same time in dream to a number of individuals. Even upon the supposition that all the soldiers were drawn from the same district, and that in Auvergne the recognised form of Satan was canine and of the Catholic colour black, there will remain an unexplained difficulty, not more inexplicable, however, than many phases of waking thought.

The principal objects of M. Maury, in his recent work, are to present the relations which connect the different forms of delirium, from those which constitute dreaming to the profound perturbations of mania. He finds, however, that there are two series, or scales, the zero of which are dreamless sleep and senile dementia; but in ascending, sleep with transitory, confused

* *Gr. Dict. de Méd.* Art. "Incube," quoted by Macario, *Du Sommeil*, p. 106 et seq.

dreams is observed in the one, inconsequent ideas and incoherent language are met with in the other. The delirium of the dreamer, that of the maniac, and that of the fevered, representing respectively the healthy, the chronic, and the acute pathologic stage, or that intellectual disturbance in which the association of ideas becomes imperfect, and the sensorial hallucinations cannot be distinguished from the real impressions of the senses. Somnambulism, natural and artificial, ecstasy, hypnotism, place the economy in an analogous semi-pathological condition, as far as the intellect is concerned; as they belong to that class of disorders in which external sensations are partly abolished and partly perverted, and the weakened consciousness of which falls under the governance of spontaneous or communicated ideas, and accepts the former as strange and new, and the latter as familiar. In natural somnambulism, the process is idiopathic; in ecstasy and artificial somnambulism, it depends upon agents acting at the same time upon the mind and body; in anæsthesia, upon agents influencing first the general system, but reacting upon the brain. In intoxication from narcotics or alcohol there is primarily obtuseness of the organs of sense and sensorial apparatus, associated with cerebral excitement. All these pathological states are closely allied, present similar indications, and run into each other, proving that they do not correspond to any particular psychical state of the spirit, but that they depend upon a disorder of the nervous system, of which derangement is the culminating point. Dream is the starting-point; the elementary form of all these phenomena, as it is referable to periodic exhaustion, furnishes the first lineaments of mental maladies, is often complicated with partial excitement, and is sometimes accompanied by a passing, morbid, but accidental state of the economy. All these conditions, and the nervous disorders with which they are associated, are generally connected with a lesion, more or less extended, of innervation. Pathologically they differ. The same delirium may proceed from many different alterations of the nervous system and encephalon. Sometimes there is mere congestion, slight arrestment of the cerebral circulation, as in sleep; sometimes excitement in one portion, congestion in another, as in sleep with distinct and multiplied dreams; sometimes exaltation of certain fibres, such as to destroy the action of others, as in somnambulism; sometimes acute congestion of the brain, as in intoxication and the delirium of fevers; sometimes organic alteration of parts of the nervous substance, as in mania and dementia; sometimes paralysis, either by compression of the brain by blood, or by enfeeblement of the nerve-force itself, as may be seen in certain kinds of insanity and hypnotism. When a man sleeps—that is to say, from the moment that his functions begin to lose their essential activity—

intellect sustains irregularity of action, which is repeated, continued, extended, multiplied into irregularities, which speedily produce that series of derangements and perversion which eventuate in fatuity and death. It is in this aspect, and in this only, that dreams ought to be associated psychologically with alienation. There is a common parentage of all species of delirium, and that is an unhealthy condition of the cerebrum, which consists in a state of congestion; but besides this, the automatic movements and the excitation of the senses which determine hypnogogic hallucinations, continue during sleep, and are the active factors of dreaming. M. Maury speculates often very hypothetically as to vibrations of nervous fibres and spasmodic action, and concludes, "Mais le sommeil est l'image de la dégradation intellectuelle et de la perte de l'intelligence;"* yet, in the further discussion of the question, bears apparently unwilling testimony that "le songe, comme le corps des idées de l'enfant, est en majeure partie composé de ses souvenirs ignorés."†

The tendency to establish intimate relations between dreaming and mental disease, and other conditions, such as artificial somnambulism, which are anormal if not morbid, and to demonstrate that these are merely degrees of the same unhealthy action; and the attempts, especially by the physiological school, to show that the mind during sleep obeys laws differing essentially from those by which it is regulated when vigilant, have suggested the incorporation with our own remarks of occasional notices of the two works by Maury and Macario, in many respects most valuable monographs, but in which these errors run like a trap-dyke through a fertile country. A large portion of each is devoted to somnambulism, ecstasy, &c., which the authors regard as the developments or connate states of dream; and with collateral disquisitions;—but here the incidental allusions made have been confined to theses on the phenomena of dreaming, in which the supposed chain from the delirium which constitutes our dreams to the incoherence of insanity according to Maury, and on that duality of man, and irregular, divided activity of the faculties, according to Macario, which, in our apprehension, assail the common sense, and therefore the philosophical, view of the action of mind during sleep. Avoiding all attempts to penetrate into the ultimate condition of the nervous matter, and to pronounce dogmatically, as the most recent inquirers have done, that there must exist atony, or the vibration of certain fibres, and the repose of others, during sleep; the sound view appears to be that dreaming is due physically to the alternate, or co-ordinate, or irregular activity of the different portions of the brain connected with the manifestation

---

* *Op. cit.*, p. 377. † *Op. cit.*, p. 398.

of different mental faculties or feelings; that if the whole mass acts nearly as under ordinary circumstances, the dream will resemble the waking thoughts; that if portions connected with the instincts and passions are excited, there may be nightmare; that if there be want of harmony as to time, or force, or sequence, between the different parts, incoherence may ensue. There cannot, however, be any separate or independent action of different parts or powers. The mind acts in unity and solidarity in sleep as at all times; in other words, although the acts may be successive as to order, to our apprehension colours, forms, sounds, and the conceptions that they suggest, are present simultaneously and harmoniously. This is not the doctrine of the phrenologists only. We find Dr. Cappie supporting this view chiefly upon physiological grounds.*

"We thus see that in the production of sleep there is a chain or combination of physiological conditions. The first link is a modified form of nutrition in the substance of the brain; the last is pressure exerted on the surface of the organ by the veins becoming distended with blood. The principal intervening link is the diminished vascularity in the nervous tissue. No one of these conditions can be regarded as the cause of sleep. All are necessary."—p. 90. "When we reflect upon the numerous functions to which the brain is subservient, it appears certain that there must be a subdivision of labour among different parts of its mass. It is extremely improbable, then, that every part of the nervous substance of the brain will be equally affected by every physical operation. Whatever be the mode in which mental activity may manifest itself, some portions of the organ will, for the time being, be more vigorously exercised than others. When the mind is engrossed with a present sensation, or the contemplation of external phenomena, the nervous substance will be differently affected than when past impressions are recalled, or when the mind is disturbed by emotion, or the reason is adapting means to ends, or the volition is calling into play a particular class of muscles. Assuming, then, that the brain is functionally active, this activity is more or less circumscribed; that in healthy mental activity the whole organ is never equally exercised; we may also assume that the immediate seat of activity must have its vascularity increased. The arguments already advanced to prove that the brain, as a whole, must contain a larger quantity of blood during the period of wakefulness than when its functions are suspended, apply with equal force to limited portions of the organ. . . . Some of the immediate consequences of any alteration that may take place will be at once perceived. In the first place, no sooner will one portion of the brain become more vascular, than some other part must become less so. The more fully the blood is determined into the anterior cerebral arteries, for example, the smaller

* *Essays on Mental Science. On the Encephalic Circulation, and its relation to the Physiology of the Brain.* By James Cappie, M.D. Edinburgh. 1859.

must be the amount transmitted through other vessels. . . . . Secondly, a certain amount of pressure must be exerted on the surrounding tissue."—(pp. 91—93.)

It is not necessary here to point out how much of this theory is founded upon sheer assumption, because there is reason to doubt the accuracy of the broad and unqualified affirmation made as to increased vascularity, at least during sleep.

"Mr. Durham defines sleep to be that state of cerebral repose which is necessary to repose, which is essential to the nutritive repair of the brain substance, a state, therefore, in which consciousness, sensation, and volition, the great manifestations of the brain's activity, are temporarily suspended, but so suspended as to be easily restored by the action of stimuli, external or internal. By experiments on animals and ocular examination of their brains under different degrees of excitement, and during sleep, sufficient evidence has been obtained that every variation in functional activity and repose is characterized by a change in the cerebral circulation. The removal of portions of bone by the trephine, and the substitution of watch-glasses, renders the surface of the brain easy of observation. It appears from observations thus conducted that during sleep the brain is comparatively bloodless, the veins are *not* distended, and the capillaries contain little corpusculated blood. . . . . The hypothesis that venous pressure is the cause of sleep is thus shown to be untenable."*

If it be true that the mind acts in dream in the same way as when vigilant,—less the contributions and corrections of the external senses and the consequent diminished intensity of attention and consciousness,—we should expect to obtain the same kind and nearly the same amount of assistance in the examination of the laws of mind, and in the diagnosis of mental diseases from observations in this state as under ordinary circumstances. What is wanted in the prosecution of the subject is not so much facts, as a principle upon which these might be systematized; but this might be obtained by comparing the condition and dreams of different classes of the sane and insane generally, when asleep and while subjected to different external circumstances. The inability to sleep and to pass into dream is the first characteristic to be noticed. To the healthy man vigilance is an intimation of perilous activity and a forerunner of mental disease; in the maniac it is followed by dementia. This supports the view that there is something more restorative in sleep than mere rest. A very large number of hypnotics which do not act proximately or psychically, act in one, or other, or in all the following ways, which, however, approach the confines of psychogenesis, if they do not touch them. They allay pain wherever it may be situate;

* Durham, quoted in Ranking's *Abstract*, vol. xxxiii. p. 302.

they obtund the external senses and lessen their acuteness in removing and transmitting impressions; they arrest, or greatly modify, the processes of nutrition and disintegration of tissues, and consequently diminish the number and force of the reports of cœnesthesis; and they necessarily and consequently moderate and modify those molecular and other changes in the nervous matter of the brain with which psychical action, whether under the name of sensation, or the noblest sentiment, or a dream, is associated. The most celebrated psychical remedies, again, for sleeplessness may be classed as monotonous, monoideaous, or repetetive; and may be epitomized as consisting in the withdrawal of all impressions save those that can be conveyed singly, in succession, or rhythmically. From Boerhaave's dropping of water, to the Abracadabra of the secret-mongers, and the discovery of Mr. Gardener, they are involved in Cullen's anticipatory dogma, that "if the mind is attached to a single sensation, it is brought very near the state of the total absence of impressions."*

All physical. morbid conditions are attended by distressing dreams, but it would be rash to affirm that they are invariably characteristic either of the nature of the affection, or of the place affected. It has been observed that patients labouring under disease of the heart think and dream of falling from heights and precipices; under hydrothorax, of drowning; under dropsy, of allaying thirst; under fever, of fires; under cancer, of sorrow and suffering and local injury; and cases have occurred in which frequently recurring dreams of such kinds have led to the suspicion of the existence of disease when other evidence was wanting. That such correspondence, if it exists, must depend upon one of three causes, the quality of the blood circulating in the brain, the impressions conveyed to the brain through the nervous system, and upon the condition of the brain itself, is obvious. That condition must be influenced by increased or diminished density; by the state of the membranes and the bloodvessels, and by a thousand other circumstances which, apart from remote changes or blood diseases, induce unhealthy dreams. An atheromatous state of the arteries, for example, as well as alterations in the structure of the ear, is accompanied by delusions or dreams, in which sound, voices, footfalls, thunder, the gallop of horses, play a conspicuous part. Similar effects are observed in choreamania and melancholia, where anæmia is the most marked pathological state. It is interesting that in the former disease the waking mentalization and the hypnogogic phantasmata are connected with great want of attention and irregularity of thought, as if in keeping with the muscular agitation and restlessness,—

* Binns. *The Anatomy of Sleep*, p. 358.

whereas the dream of complete sleep, when the muscular system is at rest, is calm and placid. Maury has observed that there is often entire oblivion of dreams, during the continuance of which there was great muscular activity and loquacity; but in many cases night-talking and crying are signs of irritation and horror. They may accompany the night seizures of Marshall Hall. Occasionally they are so dreadful and so dreaded that the sufferer endeavours to escape by preventing sleep. It is at this point that dreams may pass into hallucinations. The mind has given way first during dream when discipline and control were removed, and the fact of its overturn has been noted by the patient;—and it has happened that the dreams which prevailed during insanity have reappeared after the re-establishment of health.

